# Do Authentic People Care about the Environment? A View from Two Paradigms

**DOI:** 10.11621/pir.2021.0306

**Published:** 2021-09-30

**Authors:** Sofia I. Reznichenko, Sofya K. Nartova-Bochaver, Boris D. Irkhin

**Affiliations:** a HSE University, Moscow, Russia

**Keywords:** authenticity, pro-environmental behavior, person-centered psychology, subjective psychology, gender, Moscow Authenticity Scale, Ecological Lifestyle Scale

## Abstract

**Background:**

Personal authenticity, the ability to be true to oneself, is traditionally studied from the perspective of its protective role for the individual and is only beginning to be studied in relation to the surrounding world. In this study, we suggest that authentic people may be more aware and concerned about their environment then less authentic people. The theoretical foundations for our work were: the person-centered approach; subject psychology; and modern research on pro-environmental behavior.

**Objective:**

We presented our understanding of personal authenticity within Russian subject psychology, developed the standardized instruments necessary for carrying out our main aim, and explored the links between authenticity and pro-environmental behavior in both person-centered and subject psychology.

**Design:**

Four hundred thirty **(**430) Russian students (M_age_ = 19.19; SD_age_ = 1.22; 79.5% women) participated in the study. Authenticity was measured both bythe revised Russian version of the *Authenticity Scale*, and a new tool, the *Moscow Authenticity Scale*, which was developed on the basis of subject psychology. To measure pro-environmental behavior, we created a new instrument called the *Ecological Lifestyle Scale*, which included *Social Activities* and *Ecological Self-restraint* subscales.

**Results:**

Using the two new scales, the *Moscow Authenticity Scale* and the *Ecological Lifestyle Scale*, along with a modification of the *Authenticity Scale*, we found that authenticity, considered within the framework of subject psychology, provided a more nuanced picture of the relationship between personal authenticity and pro-environmental behavior than the person-centered model did. Women were more likely to exercise pro-environmental behavior than men; however, the connections between personal authenticity and pro-environmental behavior were stronger in the male group.

**Conclusion:**

Authenticity is associated with pro-environmental behavior but does not predict it accurately enough. Future research on moderating or mediating variables is suggested.

## Introduction

The famous Greek maxim “know thyself,” which represents the human aspiration for harmony and wisdom in the world through self-knowledge, has been known for dozens of centuries, and, to this day, remains one of the most demanded (and demanding) endeavors. In circumstances of high uncertainty, environmental pressure, information overload, and a fast-paced life, the natural need for knowing and being yourself draws significant attention. A human’s personal and social identity changes throughout their life with increasing pace, promoting a need for a stable, phenomenological “anchoring point” that ensures the integrity of one’s self, despite intense individual and environmental changes; this anchor is represented by authenticity.

Authenticity, originating from the Greek word *αυθεντικός* (meaning true, genuine), is a personality trait that facilitates being true to oneself. It includes one’s personality, one’s own path or calling, and the circumstances of one’s life, including space, time, and environment (Nartova-Bochaver, 2011; Nartova-Bochaver, Irkhin, & Reznichenko, 2020).

Psychologists distinguish authenticity from both intrapersonal and interpersonal perspectives (Grégoire, Baron, Ménard, & Lachance, 2014). From an intrapersonal perspective, authenticity is viewed as congruence between one’s experience and values, in relation to one’s “true” self (Strohminger, Knobe, & Newman, 2017). While it does not imply judgment, due to its subjectivity, authenticity has been found remarkably important in terms of personal well-being (Chen & Murphy, 2019); stable self-esteem (Grijak, 2017); self-compassion (Yanchenko & Nartova-Bochaver, 2020); acquiring meaning in one’s life and work (King & Hicks, 2021); and overall mental health (Hallam, Olsson, Bowes, & Toumbourou, 2006; Nartova-Bochaver, 2011; Nartova-Bochaver, Irkhin, & Reznichenko, 2020) The above-mentioned studies represent significant research on the positive role of authenticity on the self-self axis, fortifying its role as a sturdy pillar of self-development.

Interpersonal authenticity is often studied as a beneficial quality of various social interactions, such as work relationships (Reis, Braga, & Trullen, 2017), leadership (Sidani & Rowe, 2018), emotional display and response (Zloteanu & Krumhuber, 2021), and personal relationships (Josephs et al., 2019). From that perspective, being authentic means respecting social norms and being responsible for one’s decisions, virtues, and moral principles, and their effects on others. At the same time, research on the self-world axis remains scarce, and the social psychology of authenticity is still in its infancy (Ryan & Ryan, 2019). In our view, discovering the effects of personal authenticity in relation to the environment is gaining increased interest, which could provide useful insights for the ongoing discussion of human-nature relations.

Is it intrinsic for people to care for and protect nature? While the overwhelming number of reports on ecological issues forecasts an unprecedented man-made planetary crisis (Das & Horton, 2018; Watts et al., 2019; Zeppetello et al., 2020), psychological research on pro-environmental behavior is gaining the utmost importance. Multiple studies on ecological trends propose that the resolution of the oncoming challenges requires a significant change of current attitudes (Pooley & O’Connor, 2000), values (Karp, 1996), or even the whole paradigm of views regarding nature (Dietz, Stern, & Guagnano, 1998; Dunlap, 1980). These proposed changes are often viewed as an educational objective in schools and universities (Zsóka, Szerényi, Széchy, & Kocsis, 2013), based on evidence that younger people are more adaptable in their attitudes (Liefländer & Bogner, 2014) and are more focused on and motivated toward building a constructive relationship with nature.

One idea of an ecologically-minded person is represented in Carl Rogers’ vision of future human moral values. In Rogers’ view, human beings have an intrinsic potential to care for nature and be close to it (Rogers, 1995). Since his work, environmental psychology has found several nature-related phenomena that confirm his ideas of a deep link between humans and the natural world (Clayton, 2003; Mayer & Frantz, 2004). However, this link seems to be weakened by the overwhelming stress and monotony of modern life, and a lack of time spent in natural surroundings, which increases the risk of developing psychological disorders like the nature deficit disorder (Kuo, 2013). Connection to nature also appears to be gender-related. Women have consistently shown higher relatedness to nature (Lawton, Brymer, Clough, & Denovan, 2017; Irkhin, 2020) and more participation in pro-ecological activities than men (Richardson, Cormack, McRobert, & Underhill, 2016; Dietz et al., 1998; Kennedy & Kmec, 2018).

Person-oriented scholars, following Rogers, saw the appropriate human response to the planetary crisis in personal development, congruence, and authenticity facilitation (*e.g.,* Barrett-Lennard, 2013; Cornelius-White, 2006; Joseph, 2016; Neville, 2013). In person-centered psychology, a morally mature person seeks harmony, not only inside, but also around their self. When they consider nature a part of themselves, people become more concerned about the environment (Clayton, Irkhin, & Nartova-Bochaver, 2019; Clayton & Kilinç, 2013; Vorkinn & Riese, 2001), and are prone to display pro-environmental behavior (Lee & Lim, 2020; Tam & Chan, 2018).

Taking into account the rather unique Russian culture and mentality, often placed somewhere in between collectivism and individualism (Mamontov, Kozhevnikova, & Radyukova, 2014), in Russia a new model of authenticity called subject psychology was developed by the famous Soviet psychologist Sergei Rubinstein (1889-1960). Rubinstein was one of the “pillars” of Soviet psychology. He was educated in Germany and demonstrated an unusual breadth of interests and dialectical thinking. According to his theory (Rubinstein, 2012), which was influenced by the works of Franz Brentano, personality always exists in the context of the environment, circumstances, and the world as a whole. We cannot extract an individual from this context; thus, the true self necessarily includes the presence of the social and natural circumstances of their life.

In contrast to Rogers’ person-centered approach, which considers social influences as a source of violation of the individual’s authenticity, the subject approach sees the true self involving mutually productive interactions of the individual with other people, culture, and nature (Znakov, 1998; Znakov, & Sverchkova, 2003). These ideas were taken into account when we developed the *Moscow Authenticity Scale*, presented below.

Authenticity, defined as the ability to be oneself or to follow one’s true self, is considered in our study as the coherence of a person’s life experiences (actions, cognitions, and emotions), on the one hand, and his/her personality (temperament, values, beliefs) and the circumstances of his/her life (time, place, and life-calling), on the other (Nartova-Bochaver, Reznichenko, & Maltby, 2021). In contrast to the person-centered model of authenticity, which has been criticized as an individualistic one (Strohminger et al., 2017), authenticity in subject psychology acts as an integral characteristic of a person, represented by both a subjective attitude toward the self and the world around them (Slobodchikov & Isaev, 1995). Thus, authenticity appears as a holistic phenomenon, implying the inseparability of the person from the world, and their interdependence. In the prism of subject psychology, the acquisition of one’s true self is carried out through ethical attitudes and placing oneself in the context of social relations (Rubinstein, 2012). Therefore, involvement in social relationships and the establishment of honest, harmonious relationships with others, are inseparable from authentic living and self-realization.

Ottiger and Joseph (2020) revealed a significant positive connection between authenticity and ethically minded consumer behavior, providing the first empirical support for Rogers’ idea of an ecological mindset. However, when taking into account that the person-centered conceptualization of authenticity does not work very well in Russia (Nartova-Bochaver et al., 2021), it is necessary to consider an alternative understanding of authenticity, namely, one developed within the framework of Russian subject psychology.

In the current research, we examined the link between authenticity (understood from the two points of view) and pro-environmental behavior. We considered pro-environmental behavior as those actions that increase environmental benefits or reduce environmental harm (Steg & Vlek, 2009).

To investigate the possible link between personal authenticity and pro-environmental behavior, we hypothesized that:

1. Personal authenticity would positively correlate with pro-environmental behavior; and
2. This connection would be moderated by conceptualization (in the framework of subject psychology, it would be stronger than with the person-centered approach).

First, we present the preliminary psychometric work which was required to perform our main study.

## Methods

### Participants

The students took this survey as part of their homework, via the online service 1ka.si. A total of 430 people, in the age range of 17**–**26 years old (M_age_ = 19.19; Me_age_ = 19.0; SD_age_ = 1.22; 79.5% women) participated. All participants were bachelor or master’s program students from Moscow universities. Participation was voluntary; all participants gave their informed consent to anonymously publishing their data. Along with the main questionnaires, respondents provided demographic information (age, sex, ethnicity, and religion).

### Measurement Instruments

#### Authenticity Scale

The modified *Authenticity Scale* measures an individual’s self-reported authenticity trait. Based on the person-centered approach, authenticity is conceptualized as a tripartite construct comprising *Authentic Living*, not *Accepting External Influence*, and a lack of *Self-Alienation* (Wood et al., 2008) (Appendix 1). Ithas 11 items, with a seven-point Likert scale, ranging from 1 (does not describe me at all) to 7 (describes me very well).

#### Moscow Authenticity Scale (MAS)

The MAS is a new one-factor tool developed within the framework of subject psychology. It consists of five items (Appendix 2), with a five-point Likert scale, ranging from 1 (strongly disagree) to 5 (strongly agree).

#### Ecological Lifestyle Scale (ELS)

The ELS is a new tool developed to measure pro-environmental behavior as a person’s stable behavioral pattern, reflecting their eco-centric worldview. The ELS includes two subscales: *Social Activities* (three items), which describes the person’s purposeful social activities aimed at protecting nature, and *Ecological Self-Restraint* (four items), which describes routine pro-ecological actions aimed at an environmentally-friendly lifestyle. A five-point Likert scale, ranging from 1 (never) to 5 (very often), was used (Appendix 3).

### Analytical Strategy

First of all, item pools were developed for two new questionnaires, the *Moscow Authenticity Scale* and the *Ecological Lifestyle Scale.* The items were developed based on both deductive (literature review and assessment of existing scales) and inductive (exploratory research methodologies, including focus group discussions and interviews) methods. Seven experts in personality psychology and environmental psychology participated in formulating the questionnaire items.

After the experts’ deliberation, item reduction, dimensionality testing, and verification of psychometric properties (model quality and reliability) of the new questionnaires were carried out. Decisions on the reduction of items and the choice of the optimal factorial structure of the questionnaires were based on the results of Horn’s parallel analysis (both principal axis analysis and principal components), exploratory factor analyses (EFA), and confirmatory factor analyses (CFA). Both EFA and CFA were performed using the robust ML (MLR) rescaling-based estimator, due to its ability to handle ordinal variables. To assess the model fit of each of the developed questionnaires, we examined both absolute and incremental fit indices, including the Root Mean Square Error of Approximation (RMSEA) and its 90% confidence interval, as well as the *p*-value of Close Fit (PCLOSE), the Comparative Fit Index (CFI), the Tucker Lewis Index (TLI), and the Standardized Root Mean Square Residual (SRMR).

The internal reliability of all developed scales was estimated with both Cronbach’s Alpha and McDonald’s Omega.

Finally, the relationships between authenticity (the independent variable) and pro-environmental behavior (the dependent variable) in male/female groups were examined via ANOVA, the Pearson correlation, and multiple regression analysis. To address the imbalanced classification problem (20.5% male), we used weighted sampling while processing regression analyses (weights of 3.5 for the minority class) and also used bootstrap, based on 1000 samples, to check the significance level of predictors in the regression model.

Statistical analysis was performed using R Software and Programming, environment 4.0.2 (R Core Team., 2020), SPSS v. 23, and MS Excel 2016 software.

## Results

### Modification of the Authenticity Scale

A recent study (Nartova-Bochaver et al., 2021) had presented a Russian version of the *Authenticity Scale* (Wood et al., 2008). Although it was a working instrument, we decided to modify it, because the authors reported a bias towards lower scores, a potential ceiling effect, and the cognitive complexity of one item. Moreover, the subscale measuring *Authentic Living* in this version, turned out to be inverted relative to the original, and we hoped that by selecting more accurate wordings, we would be able to make it direct; unfortunately, this was not possible.

In the current study, we developed additional (“spare”) versions for five items; the resulting scale included 16 items. All previous studies devoted to the analysis of the factor structure of the *Authenticity Scale* (Wood et al., 2008; Nartova-Bochaver et al., 2021; Di Fabio, 2014), indicated that the hierarchical model (three uncorrelated first-order factors and a higher-order A*uthenticity* factor) describes empirical data better than a first-order correlated model or a bifactor one. Taking into account the high-level reproducibility of the results and reliability of the hierarchical model of the *Authenticity Scale*, and also small substantive changes in the current version of the scale, we focused on the analysis of the hierarchical model and did not compare its fit indices with other alternative models.

Based on the CFA modification indices, factor loadings, and data on item distributions, we chose the optimal three-factor model, which consists of 11 items (Appendix 1). As expected, the fit indices and internal reliability of the modified version of the model were better than in the model created during primary validation (Hu & Bentler, 1999) (*Table 1*). All items were normally distributed (ranging from –1 to 1); the factor loadings of the current model were also higher than the loadings obtained in the primary validated model: 0.57–0.91 vs. 0.53–0.79 on the first-order factors, and 0.71–0.92 vs. 0.72 to 0.89 on the second-order authenticity factor. The modified model explained 68.9% of the variance, while the primary validated model explained only 56.7%. As in the original version, the current one includes three subscales, such as *Authentic Living*, *not Accepting External Influence*, and a lack of *Self-Alienation.*

**Table 1:** Comparison of the fit statistics and reliability between Authenticity Scale versions

	Primary validation (Nartova-Bochaver et al., 2021)	Current validation
*Fit statistics*		
X^2^/(df)	2.98	1.64
CFI	0.961	0.985
TLI	0.949	0.980
RMSEA [95% CI]	0.050 [0.040–0.060]	0.039 [0.023–0.053]
PCLOSE	0.347	0.893
SRMR	0.037	0.033
*Reliability ω (∝)*		
Authentic Living	0.78 (0.64)	0.76 (0.71)
Accepting External Influence	0.79 (0.71)	0.81 (0.80)
Self-Alienation	0.84 (0.80)	0.91 (0.89)
Authenticity total	0.89 (0.84)	0.92 (0.89)

*Note. ω = McDonald’s Omega, ∝ = Cronbach’s Alpha*

### Development of the Moscow Authenticity Scale (MAS)

#### Item pool development

We aimed to design the *MAS* as a time-effective express tool which should measure personal authenticity as a holistic phenomenon in line with subject psychology. When formulating the *MAS* items, we emphasized such features of an authentic person as the acceptance of when, in the stream of time, a person has been “thrown-in” to life, the coherence of one’s personality, and one’s general life course, in accordance with our definition of authenticity. Thus, the *MAS* items reflected a synergistic, holistic model of a person-in-the-world. In contrast with the items of the *Authenticity Scale*, interaction with the social world was interpreted in terms of environmental pressure, and most wordings described manifestations of self-alienation, rather than authenticity itself.

Seven experts worked together to develop and refine the items and consulted other scholars. The pool included 12 items; then, we set a limit of 5**–**7 items as the target number of statements.

#### Factor analysis and reliability testing

A principal component exploratory factor analysis, with varimax rotation and extraction based on eigenvalues greater than 1, was conducted, from which a two-factor solution (accounting for 58.25% of the variance) converged in four iterations. Five items, with loadings lower than 0.50 and/or cross-correlations above 0.30, were dropped and an additional iteration was conducted, which resulted in a one-factor structure (explained variance = 55.30%), with loadings for the remaining items higher than 0.50. The results of Horn’s parallel analysis showed that a one-factor solution was optimal.

Further, the resulting one-factor model, with the remaining seven items, was tested using CFA. The scaled fit indices (X^2^(14) = 63.773; *p* < 0.001; CFI= 0.945; TLI = 0.917; RMSEA = 0.090 (95% CI [0.069; 0.114]); and SRMR = 0.410) were unacceptable (Hu & Bentler, 1999). To make an additional improvement of the model, we checked the modification indices (MI) with values higher than 10. The MIs suggested that the model could be improved by drawing error covariances between the items *“I live in accordance with my beliefs”* and *“Although I’m wrong***,** *I’m living my own life”*) (MI = 23.06), and the item “*I know for a fact that I am not living my life in vain,”* along with three other items (MI = 11.15–15.67).

Since there was no strong theoretical rationale to add the error covariances between the items, we decided to remove the two items with the lowest factor loadings (“*I live in accordance with my beliefs”* (0.50) and “*I know for a fact that I am not living my life in vain*” (0.51). These changes improved the model: all fit indices showed a perfect fit of the model (X^2^(5) = 3.767; *p* < 0.583; CF = 1.000; TLI = 1.000; RMSEA = 0.000 (95% CI [0.000; 0.051]); PCLOSE = 0.946; and SRMR = 0.017) (Hu & Bentler, 1999). The range of the factor loadings of the five items included in the model was 0.51–0.78; the amount of variance explained by the model was 51.8%.

Internal consistency measured with McDonald’s Omega and Cronbach’s Alpha was satisfactory: 0.767 and 0.757 respectively (Hair, Black, Babin, & Anderson, 2010) (Appendix 2).

### Development of the Ecological Lifestyle Scale (ELS)

#### Item pool development

To accomplish the main aim of the study, we needed a new instrument that would measure a person’s pro-environmental behavior and ecocentric belief system, since most of the existing tools were developed in WEIRD (Western, educated, industrialized, rich, and democratic) cultures, which differ from the lifestyle of Russians.

During the development of the item pool, some statements were borrowed from works by Markle (2013) and Clayton et al. (2021) and subsequently modified (questions were reformulated into statements according to forward and back-translation guidelines (“Process of translation and adaptation of instruments,” n.d.). As a result, the initial pool included 12 items, reflecting both environmental routine actions (*e.g.*, water and power conservation, sorting of waste, reduced car use, refusal of meat products, etc.) and civic actions (*e.g.,* prosocial behavior; volunteering for nature protection activities, and donating to environmental charities).

#### Factor analysis and reliability testing

According to Kaiser’s eigenvalue > 1 criterion, the number of factors to be retained was three (eigenvalues of 4.36, 1.47, 1.11), which accounted in total for 57.74% of the variance, but examination of the scree plot suggested only two factors. Based on Horn’s parallel analysis, a two-factor solution was optimal. During EFA, one item (“*If possible, I avoid consuming animal-based food [meat products]”*) was excluded due to its low factor loading (> 0.3).

When conducting CFA, we built a hierarchical model with two uncorrelated first-order factors and a higher-order factor — *Total Ecological Lifestyle.* Our choice of the higher-order model, rather than the correlated factors model, is explained by the fact that a person’s pro-environmental behavior is considered as an integral construct consisting of several subordinate “traits” (Markle, 2013; Clayton et al., 2021). Accordingly, the structural model should assume the possibility of calculating scores on both the subscales and the overall score, unlike the correlated factors model, which can’t incorporate any general factor. When investigating a new tool, Brown (2015) suggests using the higher-order structure rather than the bifactor model to explore theoretical understandings of the relationship between a series of subscales which are distinct from one another but united by a common factor.

The model fit values were not satisfactory; however, the two latent factors loaded highly (< 0.6) on a higher-order factor, suggesting that a hierarchical structure was appropriate to explain the relationships between the latent variables. To improve the quality of the model, four items with factor loadings below 0.50 and/ or high error covariances between the items were removed (Appendix 3). The resulting hierarchical model, with two latent factors, showed satisfactory scaled fit indices: (X^2^(12) = 11.013; *p* < 0.528; CFI= 1.000; TLI = 1.000; RMSEA = 0.000 (95% CI [0.000; 0.044]); PCLOSE = 0.977; and SRMR = 0.024) (Hu & Bentler, 1999). The first factor, labeled *Social Activities,* contained three items (participation in voting, rallies, and house meetings). This subscale had satisfactory reliability values: ω = 0.828 and ∝ = 0.812 (Hair et al., 2010). The second factor, named *Ecological Self-Restraint,* included four items regarding actions aimed at consumption reduction and a nature-friendly lifestyle. The reliability of this scale was acceptable (ω = 0.675; ∝ = 0.643) for a research instrument with few items (Taber, 2017).

The *Total Ecological Lifestyle* factor explained 0.807 of the variance at the first-order factor level (hierarchical Omega); Cronbach’s Alpha was also satisfactory (∝ = 0.746). The factor loadings on the first-order factors were reasonable (between 0.43 and 0.86). Taken together, *Social Activities* and *Ecological Self-Restraint* explained 59.7% of the variance and loaded highly on a higher-order *Total Ecological Lifestyle* factor (0.63 and 0.79 respectively).

### Main Study: the Connection between Personal Authenticity and Pro-environmental Lifestyle

First of all, we were interested in whether there were any gender differences between the means of the investigated variables. Since the assumptions of normal univariate distribution (the values for asymmetry and kurtosis were between –1 and +1) and the equality of variances (Levene Statistic > 0.05) were met, an ANOVA was carried out, which demonstrated that men and women significantly differed in the mean values of *Accepting External Influence* (F(1; 429) = 7.36; p = 0.007); *Social Activities* (F(1; 429) = 10.66; p = 0.001); *Ecological Self-Restraint* (F(1; 429) = 7.15; p = 0.008); and *Total Ecological Lifestyle* (F(1; 429) = 12.61; p = 0.000) (*Table 2*).

**Table 2:** Mean values and Standard Deviations of the Authenticity Scale, the Moscow Authenticity Scale, and the Ecological Lifestyle Scale in male & female groups, and the entire sample

	N (%)	Age	AL	AEI	SA	MAS	SoAct	Sf-R	ELS
Male	88 (20.5)	19.30 (1.73)	13.49 (4.44)	**12.85** **(6.86)**	12.15 (5.21)	18.56 (3.96)	**5.59** **(2.84)**	**11.21** **(4.27)**	**16.80** **(5.96)**
Female	342 (79.5)	19.17 (1.05)	12.76 (4.27)	**15.11** **(6.99)**	12.55 (5.24)	18.26 (3.56)	**6.82** **(3.21)**	**12.44** **(3.74)**	**19.25** **(5.74)**
Entire sample	430 (100)	19.19 (1.22)	12.91 (4.31)	14.65 (7.02)	12.47 (5.23)	18.32 (3.64)	6.57 (3.17)	12.18 (3.88)	18.75 (5.86)

*Note. Significant differences in mean values on subscales for men and women are in **bold** (p < 0.05). AL = Authentic Living; AEI = Accepting External Influence; SA = Self-Alienation; MAS = the Moscow Authenticity Scale; SoAct = Social Activities; Sf-R = Ecological Self-Restraint; ELS = total scores on Ecological Lifestyle Scale*

Next, we analyzed the correlations between authenticity and ecological lifestyle variables (*Table 3*). Weak, but significant correlations were found between the *Moscow Authenticity Scale* (r = 0.166; p = 0.000), *Authentic Living* (r = 0.100, p = 0.005), and *Self-Alienation* (r = –0.121; p = 0.001 respectively), on the one hand, and the *Social Activities* subscale on the other. *Ecological Self-Restraint* had no interrelations with the authenticity variables. Total scores on the *Ecological Lifestyle Scale* positively correlated with the *Moscow Authenticity Scale* (r = 0.136, p = 0.001) and *Authentic Living* scores (r = 0.083; p = 0.022) and had a negative relationship with the *Self-Alienation* subscale (r = –0.094; p = 0.009). The correlations between ecological lifestyle *(ELS)* variables and the person-centered tool of measuring authenticity *(MAS)* were stronger than the correlations obtained between ecological lifestyle *(ELS)* and the person-centered framework of Authenticity consideration (the *Authenticity Scale*), confirming Hypotheses 1 and 2.

**Table 3:** Correlations between MAS, Authenticity Scale, and ELS

	MAS	AL	AEI	SA	SoAct	Sf-R	ELS
MAS		0.491**	–0.670**	–0.449**	0.166**	–	0.136**
AL	0.491**		–0.593**	–0.576**	0.100**	–	0.083*
AEI	–0.670**	–0.593**		0.474**	–	–	–
SA	–0.449**	–0.576**	0.474**		–0.121**	–	–0.094**
SoAct	0.166**	0.100**	–	–0.121**		0.370**	0.792**
Sf–R	–	–	–	–	0.370**		0.860**
ELS	0.126**	0.083*	–	–0.094**	0.792**	0.860**	

Note. MAS = the Moscow Authenticity Scale; AL = Authentic Living; AEI = Accepting External Influence; SA = Self-Alienation; SoAct = Social Activities; Sf-R = Ecological Self-Restraint; ELS = total scores on Ecological Lifestyle Scale. * = p < 0.05, ** = p < 0.01, – = statistically insignificant correlations

Multiple regression analysis was carried out, using the enter method, in order to determine whether authenticity could predict pro-environmental behavior. Three subscales of the *Authenticity Scale*, and the scores on the *Moscow Authenticity Scale,* as well as gender (dummy-coded: 1 = male, 2 = female), were taken as independent variables, and the *Social Activities* and *Ecological Self-Restraint* subdomains of ecological lifestyle were taken as the dependent variables.

The regression model for *Social Activities* and *Ecological Self-Restraint* included two significant predictors: scores on the *Moscow Authenticity Scale* and gender (*Table 4*). Although technically both models were significant, confirming connections between the investigated variables, the determination coefficients of both models were low (R^2^ = 0.073 and 0.027). The scores on the *Moscow Authenticity Scale* and gender explained the variability level of ecological lifestyle subscales poorly, amounting to less than 8%. Thus, we cannot state that authenticity significantly contributed to an ecological lifestyle.

**Table 4:** Prediction model for ecological lifestyle in the entire sample (N_weighted_ = 650)

	Independent Variables	β	b	SE b	t	p (pbt)
Model 1: prediction for *Social* F(3; 647*Activities* ) = 26.482, p < 0.001, R^2^ = 0.073	Constant term		1.384	0.699	1.980	(0.05 0.06)
	Sex	0.205	1.271	0.234	5.426	< (0.0010.001 )
	MAS	0.191	0.158	0.031	5.059	< 0.001 (0.002)
Model 2: prediction for *Ecological* F(2; 647) = *Self-*9.001*Restraint*, p < 0.001, R^2^ = 0.027	Constant term		10.349	0.794	8.468	(< < 0.001 0.001)
	Sex	0.156	0.182	0.048	4.045	< (0.0020.001 )
	MAS	0.139	–0.141	0.041	2.649	0.008 (0.01)

*Note. MAS = the Moscow Authenticity Scale; β = standardized beta coefficient, b = unstandardized coefficient beta; SE = standard error; p_bt_ = bootstrapped significance level*

At the next stage, we analyzed the links between the authenticity variables and ecological lifestyle subscales in the female and male groups separately. According to the results of the regression analysis, only the *MAS* scores made a positive and significant contribution (β = 0.163; p = 0.002) to *Social Activities* in the female group. *Ecological Self-Restraint* was not associated significantly with any of the authenticity variables (*Table 5*). The significant predictor of *Social Activities* in the male group were *MAS* scores (β = 0.234; p = 0.001), and the predictors of *Ecological Self-Restraint* were *MAS* and *Authentic Living* scores simultaneously (*Table 6*). Interestingly, the *MAS* scores contributed positively to *Ecological Self-Restraint* in the male group (β = 0.321, p = 0.001), while *Authentic Living* contributed negatively (β = -0.343, p = 0.001), although these predictors were positively correlated with each other (r = 0.491, p < 0.001). These results partially confirmed Hypothesis 2.

**Table 5:** Prediction model for ecological lifestyle in the female group (n = 342)

	Independent Variables	β	b	SE b	t	p (pbt)
Model 1: prediction for *Social Activities* F(2; 340) = 9.314, p = 0.002, R^2^ = 0.027	Constant term	4.124		0.899	4.590	<0.001 (<0.001)
	MAS 0.163	0.147		0.048	3.052	0.002 (0.007)
Model *Ecological* 2: prediction *Self-Restraint* for	No variables were entered into the equation					

*Note. MAS = the Moscow Authenticity Scale; β = Standardized beta coefficient, b = unstandardized coefficient beta; SE = standard error; p_bt_ = bootstrapped significance level*

**Table 6:** Prediction model for ecological lifestyle in the male group (n_weighed_ = 308)

	Independent Variables	β	b	SE b	t	p (pbt)
Model 1: prediction for *Social Activities* F(2; 306) = 17.707, p < 0.001, R^2^ = 0.052	Constant term		3.268	0.757	1.732	0.001 (0.007)
	MAS	0.234	4.208	0.040	2.231	< 0.001 (0.002)
Model 2: prediction for *Ecological* F(3; 305) = *Self-*20.352*Restraint*, p < 0.001, R^2^ = 0.121	Constant term		9.275	1.125	8.246	< 0.001 (< 0.001)
	MAS	0.321	0.342	0.065	5.243	< (0.0040.001)
	AL	–0.343	–0.328	0.058	–5.634	< 0.001 (0.002)

*Note. MAS = the Moscow Authenticity Scale; AL = Authentic Living; β = Standardized beta coefficient; b = unstandardized coefficient beta; SE = standard error*

## Discussion

Before we could prove the main hypotheses of the study, we had to develop or modify our techniques: the renewed versions of the *Authenticity Scale* (Wood et al., 2008), the *MAS,* and the *ELS.*

The Russian version of the *Authenticity Scale* (Nartova-Bochaver et al., 2021) went through a set of adjustments. Several items were reformulated for better clarity, and five additional versions of the translation were needed to replace the previous ones, which led to better internal reliability and fit indices. Unfortunately, we were not able to keep the *Authentic Living* subscale not inverted as in the original scale.

We also developed a new short tool for assessing an authentic personality, named the *Moscow Authenticity Scale* (MAS), in light of the fact that subject psychology developed as a scientific school mainly in Moscow. The content of the subscale items reflected the main idea of subject psychology, which studies not the person and the world but the person-in-the-world. This also corresponds to the collectivistic side of Russian culture. The *Moscow Authenticity Scale* consisted of five items and one factor; the scale showed acceptable internal consistency and a perfect fit to the empirical data.

We also created the *Ecological Lifestyle Scale* (ELS), which measured pro-environmental behavior in the context of an impact-oriented approach. This scale was developed with regard to the pro-environmental opportunities, culture, and lifestyle of Russians. It consisted of seven items and had two factors, labeled *Social Activities* and *Ecological Self-Restraint.* The scale showed good psychometric qualities: fit scores and internal consistency. The *Ecological Self-Restraint* subscale’s reliability was slightly below the accepted range, but was still acceptable for an instrument with few items, and created purely for research (nonclinical) purposes (Taber, 2016).

Our main goal was to study the relationship between personal authenticity, understood in terms of the two research paradigms, and pro-environmental behavior.

We found a weak but significant correlation between both measures of authenticity and one of the two subscales (*Social Activities*) of the *ELS.* The *MAS* scores showed a strong positive correlation with *Social Activities*, whereas the corresponding subscale *Authentic Living* of Wood’s *Authenticity Scale*, did not form any connections at all. This demonstrated that an authentic lifestyle in the framework of person-centered psychology is absolutely orthogonal to the ecological lifestyle. In other words, among authentically living people, there could be both pro-ecological and anti-ecological ones. However, reverted subscales of the *Authenticity Scale, Accepting External Influence,* and *Self-Alienation*, formed clear negative connections with *Social Activities*: people who experience environmental pressure and those who are alienated from themselves, are not inclined to support an environment that is not friendly to them.

Interestingly, *Ecological Self-Restraint* did not correlate with any measures of authenticity; the question arises: why is this so?

In our opinion, the items of the *Social Activities* subscale clearly reflect the interdependence of a person with other people (s/he encourages other people to be active) and with nature (this activity is aimed at protecting the environment), which fully corresponds to understanding personal authenticity within the framework of subject psychology. By contrast, the items on the *Ecological Self-Restraint* subscale are not so unambiguous and allow, along with environmental motivation, pragmatism (for example, saving money for paying for electricity), which may not relate to the “true self ” in any way.

In addition, the items of the first subscale are obviously formulated in the “promotion” modality, whereas the items of the second one are formulated in the “prevention” modality. An authentic person, in accordance with the classical understanding, is creative and capable of productive activity. It can be assumed that, if pro-ecological items were formulated as motivational (for example, to clean one’s yard, plant a tree), then connections would be obtained. At the moment, this is speculation, but it can serve as a starting point for further research.

Based on these results, we partially confirmed our Hypotheses 1 and 2, with some qualifications: higher authenticity was connected with social activity but was not connected with conservation behavior. The results partially overlap with those obtained by Ottiger and Joseph (2020).

In the main study, we also witnessed ambiguous results based on gender. First of all, *Accepting External Influences*, and the totals of the *Ecological Lifestyle, Social Activities*, and *Ecological Self-Restraint* scores were lower in the male group. This means, in line with previous results (Irkhin, 2020; Lawton, Brymer, Clough, & Denovan, 2017), that the men were less closely connected with the outer world, compared with women. In other words, men seemed to be more individualistic (Borkenau, McCrae & Terracciano, 2013).

The next step of analyses we performed was the development of a multi-regression model. The only predictors of *Social Activities* (a subscale of the *ELS*) were gender and authenticity (*MAS*); *Ecological Self-Restraint* was predicted only by gender. Although the pro-ecological variables’ level was lower in males, men’s authenticity showed more connections with pro-ecological variables than women’s did. Two regression models of authenticity measures, aimed at predicting pro-ecological behavior, were built separately for the two gender groups, showing mixed results. In women, *Social Activities* were predicted by authenticity, measured by the *MAS*, while there were no predictors for *Ecological Self-Restraint.* In men, *MAS* positively predicted *Social Activities* and *Ecological Self-Restraint* (both *ELS* subscales), while *Authentic Living* (a subscale of Wood’s model) predicted *Ecological Self-Restraint* negatively. Thus, these outcomes also confirmed Hypothesis 2.

These results reflect an essential difference between the two models of authenticity. According to Rogers’ person-centered model, authenticity, even in the case of a morally mature person, cultivates independence from the outer world, including connections with nature and the environment. So, authenticity might contrapose self-transcendence, a value that is consistently associated with environmentalism (Lee, 2018). Indeed, if one’s motivation is to unite with nature, one is required to let go of one’s uniqueness and thus, in certain sense, one’s authenticity. So, when pro-environmental action requires a sacrifice of personal resources, an individualistic person encounters a contradiction between their own needs and the needs of a sustainable society. Resolving this contradiction egoistically (valuing their own needs over the needs of others) would be authentic for an individualistic person.

On the other hand, the subject psychology approach, promoted in the *MAS*, implies congruence between a person and society, therefore presuming that the values of a sustainable lifestyle are a part of one’s true self in the form of one’s larger purpose in life. This ideally allows one to avoid the contradiction between personal needs and the needs of a sustainable society, which, in our case, means that ecological behavior is not experienced as a sacrifice, but a means to fulfill a greater ecological goal.

Overall, the *MAS* showed higher correlations with an ecological lifestyle and was better at predicting pro-ecological behavior than the subscales of the *Authenticity Scale* of Wood et al. (2008). But in general, all the regression models had low coefficients of determination, which means that both authenticity measures were unable to accurately explain the variability of pro-ecological behavior.

## Limitations and Future Research

The current study was conducted with Moscow university students; our sample was also gender-biased (79.5% women), which further limits our ability to extrapolate the results to a larger population. Apart from extending the age range of the sample, it would be interesting to obtain data from people living in small towns and rural areas in order to examine the results with people with a more diversified experience of nature. Moreover, as our results are not easily interpreted, it would be worthwhile to consider other variables, and to build more complicated models, including moderating and mediating effects. We should also investigate the correlations between specific items in the *ELS*, to see which particular pro-environmental activities would correlate with authenticity. Another challenge that we set ourselves is to conduct and describe a more complete validation study of the *MAS* and *ELS*, including cross-validation and measurement invariance across age and gender. Finally, we can use (or develop) more instruments to measure environmentally friendly attitudes and behaviors.

## Conclusion

The results of the current study provide some insight into the growing research on personal authenticity and its benefits in relation to the environment. While other research has suggested a connection between authenticity and pro-environmental values (Ottiger & Joseph, 2020), the current research brought mixed results. Generally, authenticity is associated with pro-ecological behavior. However, it does not accurately predict it, which motivates us for future study of the connection, in search of possible mediators.

In comparison to Wood’s *Authenticity Scale* (2008), the *MAS* showed stronger correlations with an ecological lifestyle, especially with the *Social Activities* score, and therefore is proposed as a more accurate instrument in light of the specific Russian mentality and cultural nuances.

Results of the current study indicate that women are more likely to exercise pro-ecological behavior than men (while showing no significant difference in authenticity between genders), but the connections between authenticity and pro-ecological style were more nuanced in males.

As a side but very significant outcome, the current paper introduced three freshly developed scales necessary to conduct the main study: the revised Russian version of Wood’s (2008) *Authenticity Scale;* the *Moscow Authenticity Scale;* and the *Ecological Lifestyle Scale.* These scales show good psychometric properties and are valid for measuring authenticity and pro-environmental behavior in a Russian youth sample and can be recommended for use in various areas of non-clinical research and practice.

## Appendix 1

**Шкала аутентичности**

**The Authenticity Scale**

**Instruction**

***In Russian:***

Пожалуйста, прочтите список приведенных утверждений и оцените их с точки зрения того, насколько они характеризуют Ваши привычки и поведение. Поставьте галочку в ячейке под тем ответом, который подходит Вам.

***In English:***

Please read the list of statements provided and rate them in terms of how they characterize your habits and behavior. Please check the answer that best describes you.

**Table:**

Russian wording	English wording
1. Обычно я делаю то, что говорят мне другие люди* (*ПВВ*)	I usually do what other people tell me to do* (*AEI*)
2. Я не знаю, что я чувствую на самом деле (*СО*)	I don’t know how I really feel inside (*SA*)
3. Мои поступки и взгляды меняются в зависимости от мнения других (*ПВВ*)	I am strongly influenced by the opinions of others (*AEI*)
4. Я считаю, что должен(а) делать то, чего от меня ждут другие (*ПВВ*)	I always feel I need to do what others expect me to do (*AEI*)
5. Окружающие очень сильно влияют на меня (*ПВВ*)	Other people influence me greatly (*AEI*)
6. Мне кажется, что я не знаю себя достаточно хорошо (*СО*)	I feel as if I don’t know myself very well (*SA*)
**7. Мне не всегда удается отстоять то, во что я верю (*АЖ*)**	**I do not always succeed in upholding what I believe in (*AL*)**
**8. Не могу сказать, что я всегда бываю верен себе (АЖ)**	**I can’t say that I am true to myself in most situations (AL)**
9. Мне трудно разобраться в себе* (СО)	I find it hard to understand myself* (SA)
**10. Бывает, что мне нелегко соответствовать своим ценностям и убеждениям* (АЖ)**	**Sometimes I find it difficult to live up to my values and beliefs* (AL)**
11. Мне бывает сложно понять, кто же я такой* (СО)	It can be difficult for me to understand who I am* (SA)

*Note. Reverted items are in bold. * = new items added instead of items included in the primary validated model (Nartova-Bochaver et al., 2021); Which scale the item belongs to is indicated in the brackets: AL = Authentic Living (АЖ = Аутентичная жизнь) AEI = Accepting External Influence (ПВВ = Принятие внешнего влияния) SA = Self-Alienation (СО = Самоотчуждение)*

Responses were recorded on a seven-point scale, which ranged from 1 (does not describe me at all; не относится ко мне вообще) to 7 (describes me very well; полностью относится ко мне).

## Appendix 2

**Московская шкала аутентичности**

**The Moscow Authenticity Scale**

**Instruction**

***In Russian:***

Пожалуйста, оцените приведенные ниже утверждения с точки зрения того, насколько Вы согласны с ними. Оцените каждое утверждение на шкале от 1 (совершенно не согласен) до 5 (совершенно согласен)

***In English:***

Please rate the statements below in terms of how much you agree with them. Rate each statement on a scale from 1 (strongly disagree) to 5 (strongly agree).

**Table:**

Russian wording	English wording
1. Меня устраивает место и время, в которое мне довелось жить	1. I am satisfied with the place and time in which I happen to live
2. Я принимаю себя таким(ой), какой(ая) я есть от природы	2. I accept myself as I am by nature
3. Я знаю свое предназначение и следую ему	3. I know my calling and I follow it
4. Пусть я ошибаюсь, я проживаю свою собственную жизнь	4. Although I’m wrong, I’m living my own life
5. Я себя знаю и хорошо понимаю	5. I know and understand myself well

## Appendix 3

**Экологический стиль жизни**

**The Ecological Lifestyle Scale**

**Instruction**

***In Russian:***

Оцените, насколько часто Вы совершаете действия, описанные ниже. Оцените каждое утверждение на шкале от 1 (никогда) до 5 (очень часто).

***In English:***

Please rate your activities described below by rating each statement on a scale of 1 (never) to 5 (very often).

**Table:**

Russian wording	English wording
1. Я стараюсь собирать мусор раздельно *(ПС)*	1. I try to separate my garbage *(ESR)*
2. Я стараюсь не использовать пластиковые пакеты, совершая покупки *(ПС)*	2. I try not to use plastic bags when shopping *(ESR)*
3. Я экономлю воду и/или электричество в моём доме *(ПС)*	3. I conserve water or energy in my home *(ESR)*
4. Я голосую за проекты, связанные с местной экосистемой *(СЭ)*	4. I voted to support a policy or regulation that affects the local environment *(SA)*
5. Я подписываю петиции, касающиеся экологических проблем *(СЭ)*	5. I signed a petition about an environmental issue *(SA)*
6. Я жертвую деньги благотворительным организациям, защищающим животных и растения *(СЭ)*	6. I contributed money to an environmental, conservation or wildlife protection group *(SA)*
7. По возможности я избегаю пользоваться автомобилем, предпочитая добираться пешком, на велосипеде или общественным транспортом *(ПС)*	7. If possible, I try to walk, cycle, or take public transport instead of driving *(ESR)*

*Note. SA = Social Activities (СЭ= Социальный экоактивизм) ESR= Ecological Self-Restraint ESR = ПС = Проэкологическое самоограничение*
